# Comparative Evaluation of the Effect of Foliar Nano-Se/Si Supply on the Yield and Nutritional Quality of Iceberg and Romaine Lettuce Grown in Hydroponics

**DOI:** 10.3390/plants15152289

**Published:** 2026-07-26

**Authors:** Nadezhda Golubkina, Mikhail Fedotov, Andrey Alpatov, Andrew Koshevarov, Marina Antoshkina, Viktor Kharchenko, Otilia Cristina Murariu, Hicham Fatnassi, Gianluca Caruso

**Affiliations:** 1 Federal Scientific Vegetable Center, Moscow Region, Moscow 143072, Russia; 2 Baikov Institute of Metallurgy and Material Science, Russian Academy of Sciences, Leninsky pr., 49, Moscow 119334, Russia; 3 Department of Food Technologies, ‘Ion Ionescu de la Brad’ Iasi University of Life Sciences, 700490 Iasi, Romania; 4 INRAE PACA Research Center, 84914 Avignon, France; hicham.fatnassi@inrae.fr; 5 Department of Agricultural Sciences, University of Naples Federico II, 80055 Naples, Italy; gcaruso@unina.it

**Keywords:** *Lactuca sativa* L., selenium, silicon, nanoparticles, antioxidants, minerals, storage

## Abstract

Hydroponic production of vegetables is beneficial for plant yield and quality, especially when utilizing growth stimulators. To evaluate the effect of Se/Si nanoparticle application, two contrasting lettuce types of relatively low (Iceberg) and high (Romaine) nutritional density were foliar-treated with nanoparticles of Se (25 mg L^−1^) and Si (14 mg L^−1^), singly or jointly. Only single nano-Se/Si supply showed a beneficial effect on lettuce yield, photosynthetic pigments, and antioxidant parameters. Compared to Romaine, Iceberg displayed a lower yield increase under nano-Se/Si supply, despite the higher photosynthetic pigment rise, while Romaine lettuce was characterized by a significantly higher augmentation of ascorbic acid, polyphenols, and total antioxidant activity (AOA). Nano-Se/Si halved the chlorophyll a/b ratio in Iceberg, with no effect on Romaine lettuce. Iceberg showed more evident mineral profile changes under all Se/Si treatments, with a significant Mn increase and Fe decrease. In both lettuce types, single nano-Se supply reduced Cu accumulation by more than twice, while joint Se/Si treatment significantly diminished both Cu and Zn. Nano-Si supplementation had contrasting effects on Cu, Zn, and Mn accumulation, increasing the assimilation capacity of minerals in Iceberg and decreasing Cu and Mn levels in Romaine, with no significant effect on Zn. Under single Se supply, both lettuce types provided 920–1030 µg Se kg^−1^ d.w., compared to lower values (611–720 µg kg^−1^ d.w.) associated with joint Se/Si treatment. The results of this research confirm the existence of significant genetic peculiarities in plant adaptation connected with photosynthetic pigment accumulation and mineral profile changes in the Iceberg genotype and with antioxidant parameters in Romaine lettuce.

## 1. Introduction

The controlled conditions in hydroponics foster efficient year-round vegetable production, with significantly higher yield and quality compared to conventional greenhouse farming; reduced utilization of water, fertilizers, and pesticides; and minimal environmental pollution [1,2]. The mentioned approach is especially important for high-latitude agricultural areas, allowing for excluding long-haul transport, thus decreasing the leafy green quality loss during storage. Furthermore, a soilless system provides interesting opportunities for obtaining vegetable-derived functional food with strictly regulated biochemical characteristics highly valued for human health [3,4]. In this respect, biofortification technology with selenium (Se) was developed for basil [5] and spinach [6] production in hydroponics, while that with Si was used on green beans [7]. The described aspects draw special attention due to the intense growth stimulation effect and appreciable improvement in plant antioxidant status and adaptability [8,9], as well as an often-recorded synergetic effect on plant yield and quality [10]. Both elements are known to extend the shelf life of hydroponically grown lettuce [11,12] and protect plants against viral diseases [13,14] and environmental stresses [15,16], being active at extremely low concentrations [17,18]. The essential role of Se for humans and widespread Se deficiency in the world, and the synergism of this element with natural antioxidants [19] provide additional stimulus for Se utilization in year-round production of vegetables [8]. According to the literature reports, the efficiency of Se/Si supply may greatly vary depending on the chemical form and dose of these elements, as well as the stress level and genetic peculiarities of the plant [20,21], whose response is difficult to predict.

Recent investigations have indicated promising aspects of Se-nanoparticle application, either as an efficient biostimulant or the least toxic form of Se derivatives [17]; the nano-Si supply reportedly improves the yield, quality, and resistance of plants to biotic and abiotic stresses [22], with foliar treatment being more efficient compared to soil/nutrient solution supply [17,18]. The high permeability and lower toxicity of nanoparticles (NPs), compared to ionic forms of these microelements, as well as the enhancement of nutrient accumulation in plants due to NP supply, have elicited intensive investigations of their efficiency in crop production [23,24].

However, to date, the comparative evaluation of single or joint nano-Se/nano-Si application efficiency on plant growth and development has been poorly investigated, with a lack of comparisons in the soilless growing of leafy species, which are among the most popular crops managed with this cultivation system. In particular, hydroponic lettuce is one of the most widely consumed leafy green products in many countries worldwide [1], and the least studied under nano Se/nano Si treatment. *L. sativa* includes various types with different shapes and nutritional properties. Specifically, Iceberg and Romaine, often chosen for hydroponic production, greatly differ from each other by their biochemical and mineral characteristics, such as dry matter content, levels of chlorophyll, antioxidants, and mineral profile [25,26]. A distinctive feature of Romaine lettuce is its significantly higher nutritional density of most biologically active compounds and minerals, compared to Iceberg plants.

The present research aimed at assessing the varietal differences in Iceberg and Romaine lettuce in response to single and joint foliar Se/Si nanoparticle application in hydroponic controlled conditions.

## 2. Results and Discussion

### 2.1. Yield and Growth Indices

Plant treatment with Se or Si nanoparticles at the concentrations of 25 mg Se L^−1^ and 14 mg Si L^−1^ led to a significant enhancement in lettuce yield, with the highest beneficial effect of Se nanoparticles (Table 1). Indeed, taking into account the significant differences between lettuce types in the dry matter content, nano-Se supply resulted in yield increases per dry weight by 46.0% in Romaine lettuce and 14.6% in Iceberg, compared to the control. On the contrary, no significant effect on plant yield was recorded under joint Se/Si supply, whereas nano-Si treatment increased only the plant yield of Romaine lettuce (by 33%). The results obtained upon joint Se/Si supply do not confirm the previous discovery of synergism between the two elements in rice [27], which predominantly occurs under stress conditions not connected with closed soilless system.

### 2.2. Photosynthetic Pigments

Regarding the photosynthetic pigment accumulation in Iceberg and Romaine leaves, a positive correlation was recorded between chlorophyll and carotene levels in the control plants, which intensified upon Se/Si nanoparticle supply. Indeed, the total chlorophyll content in the leaves of Romaine control plants was 2.69 times higher compared to Iceberg lettuce, with a 3.6 times higher level of carotene (Table 2). On the contrary, compared to the control, chlorophyll increase following nano-Se and nano-Si supply was significantly higher in Iceberg plants by 1.41 and 1.95 times, respectively, than in the Romaine type (by 1.15 and 1.29 times, respectively). A significant carotene increase was recorded only in Iceberg lettuce treated with nano-Se or nano-Si, by 2.75 and 1.25 times, respectively, compared to the control. Furthermore, an extremely low effect of joint Se/Si supply on chlorophyll/carotene accumulation was detected in both lettuce types. A similar inefficacy of joint nano-selenium/silicon supply on photosynthetic pigment accumulation was previously reported in *Artemisia annua* [28], contrary to wheat grown under increased salinity showing significant photosynthesis enhancement [29]. The mentioned outcomes indicate the importance of both environmental conditions and genetic peculiarities in plant response to Se/Si treatments.

Furthermore, despite the significant increase in both chlorophyll a and b content in leaves of the two lettuce types following single Se/Si supply, only Iceberg plants showed a 2-fold decrease in the chlorophyll a/b ratio in these conditions, compared to control plants. In Iceberg leaves, a higher chlorophyll b accumulation, compared to chlorophyll a (2.03 vs. 1.13 times), indicates an increased ability of plants to absorb light around 450 nm under single Se/Si nanoparticle supply, a light wavelength that is not efficiently absorbed by chlorophyll a. The results obtained in the present research suggest that chlorophyll b is the main target of the chosen nanoparticles in Iceberg plants, considering that this chlorophyll is highly important in increasing light harvesting, is closely related to light-harvesting complexes (LHCs) [30], and stabilizes chlorophyll-binding proteins [31].

Chlorophyll b is supposed to provide higher advantages than chlorophyll a, due to its ability to harvest a wider range of light as a result of the different absorption spectrum, transferring blue–violet and orange light energy to chlorophyll a and forming stronger coordination bonds with proteins in light-harvesting complexes [30,32,33]. The detected decrease in the chlorophyll a/b ratio under nano-Se or nano-Si supply was demonstrated in other plant species, thus indicating an exclusive physiological change in Iceberg under Se/Si nanoparticle treatment. The utilization of different concentrations and other supplying methods (nutrient solution and seed priming) of Se/Si, as well as various lettuce cultivars, may help to unveil the mentioned mechanisms.

### 2.3. Antioxidant Status

According to the literature reports, Iceberg lettuce contains twice lower levels of ascorbic acid compared to Romaine plants [34]. In this respect, the present results showed significantly higher beneficial effects of Se/Si nanoparticles on ascorbic acid biosynthesis in Romaine leaves, which increased by 1.3–1.6 times compared to the 1.2–1.4 times enhancement in Iceberg plants (Table 3).

**Figure 1:**
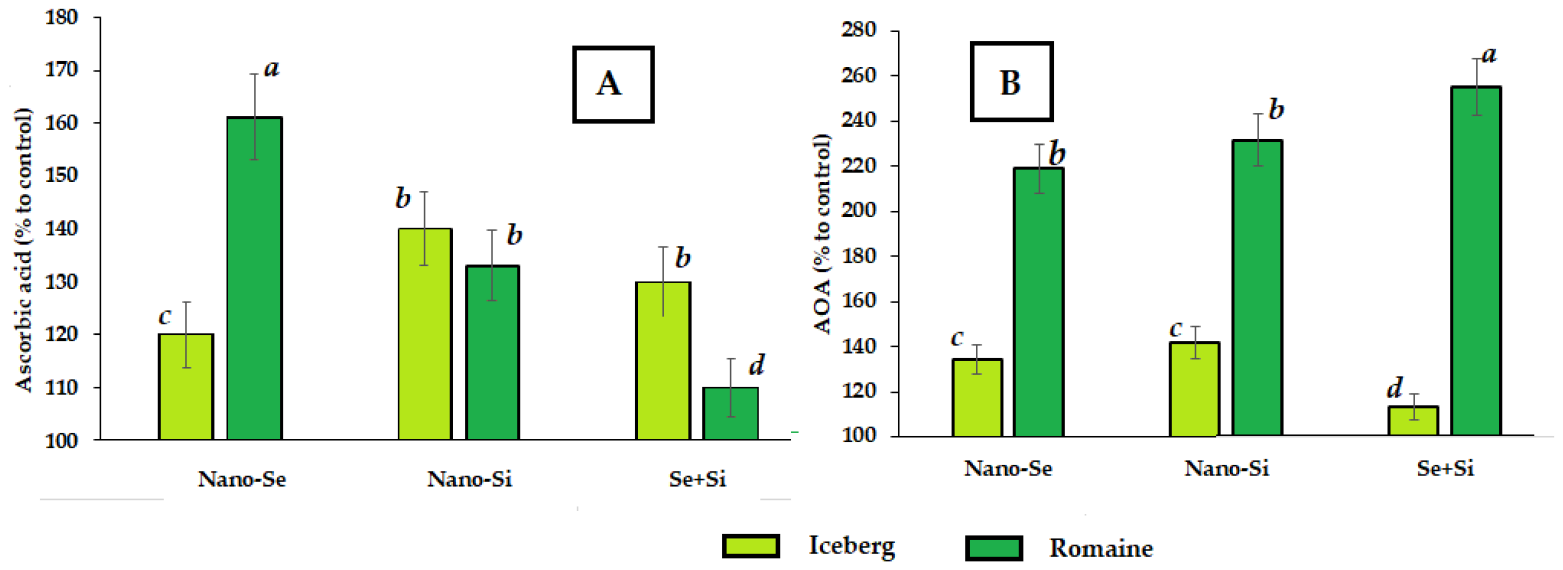
Antioxidant status in lettuce leaves: ascorbic acid (**A**); total antioxidant activity AOA (**B**). Values with the same letters do not differ statistically according to Duncan’s test at *p* < 0.05.

Even greater changes were recorded for the total antioxidant activity (AOA) in Romaine leaves, indicating a high efficiency of Se/Si nanoparticles in improving the antioxidant status of Romaine plants, with reference to both water-soluble (ascorbic acid) and ethanol-soluble (AOA) antioxidants (Table 3 and Figure 1).

**Figure 2:**
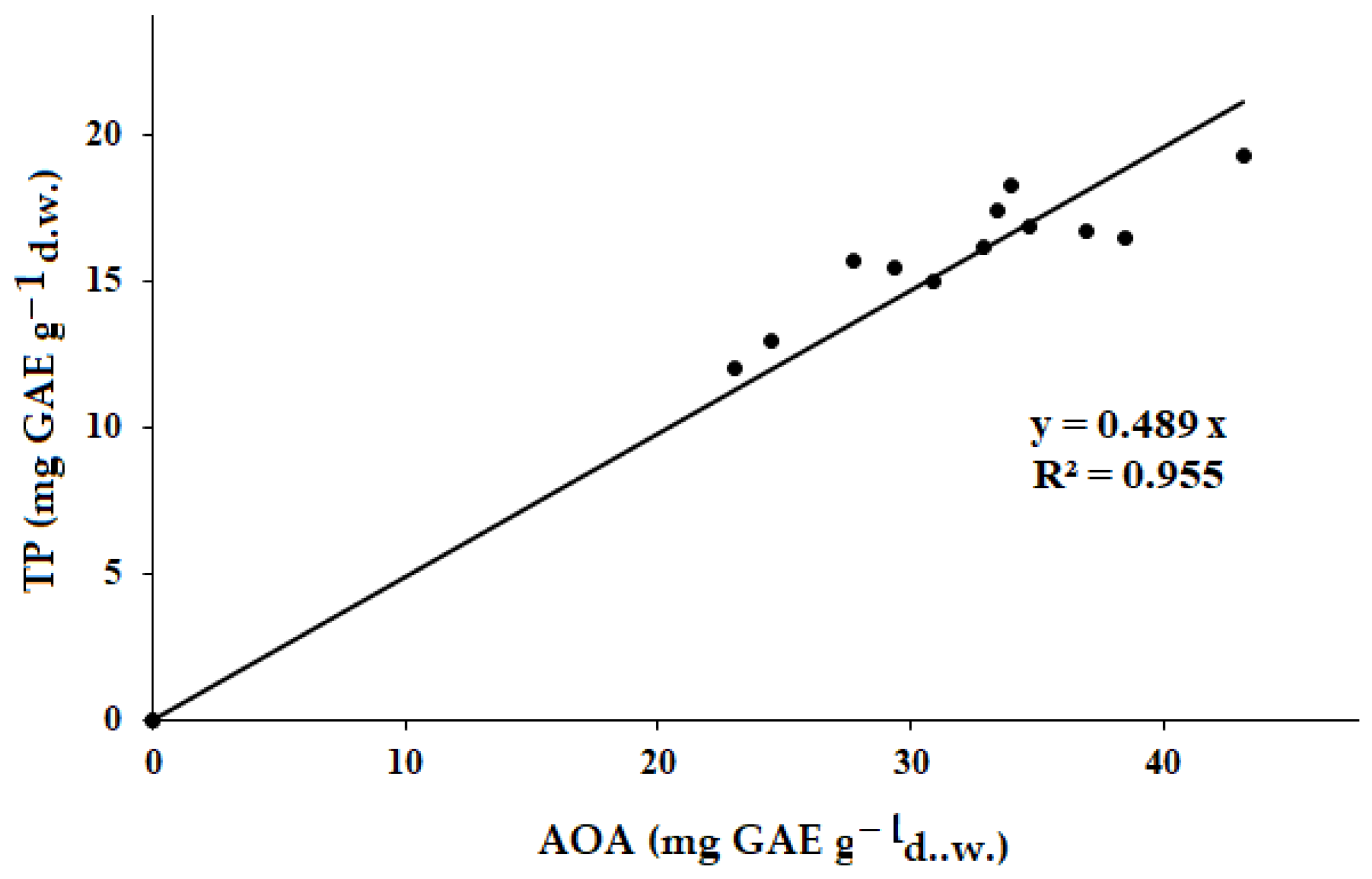
Correlation between the total antioxidant activity (AOA) and total polyphenol content (TP) in lettuce plants (r = 0.977; *p* < 0.001).

Despite the positive correlation between AOA and TP (Figure 2), it is worth noting that changes in TP were much less pronounced than AOA variations. The AOA/TP analysis included an hour of heating the 70% ethanolic extract of the leaves at 80 °C, which results in the destruction of the ascorbic acid and, therefore, excludes any effect of the latter on the AOA value.

Among the lettuce antioxidants soluble in ethanol, sesquiterpenes provide a more intensive bitter taste in Romaine compared to Iceberg, and, in general, in lettuce after storage [35]. Further investigations on the effect of nano Se/Si application on the lettuce antioxidant profile are needed to confirm the above hypothesis of sesquiterpene changes due to nanoparticle supply.

In general, the detected beneficial effects of single Se supply on lettuce yield and quality were in accordance with the main physiological role of Se in protecting plants against biotic and abiotic stresses, growth stimulation, and antioxidant status enhancement via stimulation of secondary metabolite biosynthesis [36]. The latter is achieved due to (1) the substitution of S by Se with the formation of Se-proteins with different absorption capacities and biological activity; (2) the regulation of genes responsible for secondary metabolite biosynthesis (production of phenolics); (3) the activation of antioxidant enzymes; (4) changes in the gene expression responsible for phytohormone biosynthesis; (5) changes in the gene expression of protein transporters; (6) the formation of Se-containing proteins with enhanced chelating activity; and (7) an increase in photosynthesis.

Silicon shows properties similar to Se, fostering plant resistance to environmental stresses, stimulating growth, especially in stress conditions, and strengthening antioxidant defense [16,24]. The mechanisms associated with the mentioned effects are based on (1) the maintenance of hormonal balance via appropriate gene expression changes and hormonal signaling; (2) the mechanical improvement in cell walls; (3) the upregulation of defense genes; (4) an increase in lignin biosynthesis; and (5) photosynthesis encouragement via the mechanical protection of leaves, stomatal control, and chloroplast protection.

The described phenomena are in accordance with the frequently recorded synergetic effect of Se and Si, predominantly significant under stress conditions [10]. On the contrary, the antagonism between these microelements is less frequent and may be related to (1) a hormonal imbalance caused by their different hormonal directional effects; (2) their competition for the same transport channels; and (3) an indirect effect on protein transporters of a competing element [37].

It is worth mentioning that the literature reports regarding the joint Se/Si nanoparticles treatment are extremely scant and include (i) the protection of rice against Cd/Pb stress with the indication of a Se/Si ratio significance [38]; (ii) a lack of synergism between the two microelements in protecting common bean against *Alternaria* leaf spot infection [39]; (iii) significant synergism between Se and Si in strawberry grown under drought and lack of effects in control plants [40]; and (iv) a remarkable increase in peach fruit quality of control plants due to Se/Si synergism [41]. Such heterogeneous and sometimes controversial results indicate the complexity of plant elemental homeostasis, which would require further research to unveil the mechanisms of joint Se/Si effect on plant growth regulation.

### 2.4. Storage Characteristics

**Figure 3:**
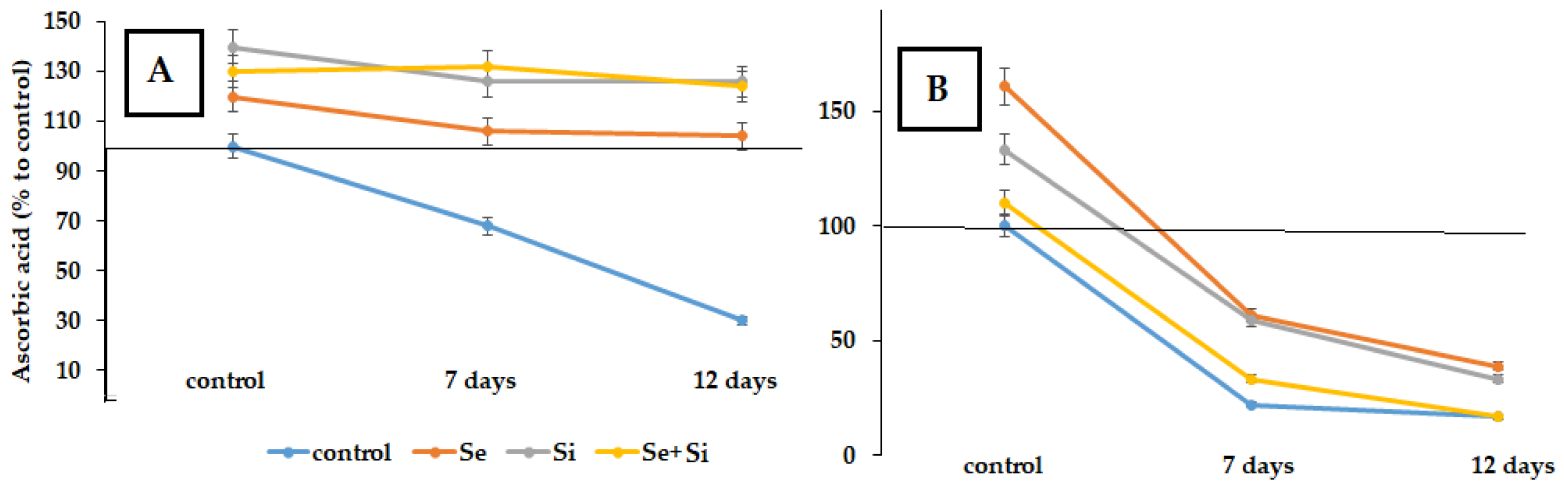
Dynamics of the ascorbic acid content in lettuce leaves during storage: (**A**) Iceberg; (**B**) Romaine.

Among biochemical characteristics of leafy green species, ascorbic acid is the least stable compound, fast decomposing during storage and, therefore, is the most important storability indicator, especially valuable for lettuce plants. The obtained results of lettuce storage at 4 °C revealed significant varietal differences in the mentioned parameter (Figure 3). Indeed, in conditions of extremely low ascorbic acid content in the Iceberg control plants, the Se/Si treatment enabled the plants to retain an initial low concentration of vitamin C for 12 days of storage in a refrigerator, with the highest beneficial effect following a single application of nano-Si or in combination with nano-Se (Figure 3A). On the contrary, nano-Se/Si supply to Romaine plants led to a significant improvement in ascorbic acid stability during this period; this occurred exclusively under single Se/Si application, with no significant effect of joint nanoparticle treatment compared to control plants (Figure 3B).

The detected phenomenon of higher ascorbic acid degradation rate in Romaine lettuce leaves during storage is presumably connected to the significantly higher levels of Fe and Cu capable of catalyzing vitamin C oxidation [42,43].

### 2.5. Mineral Composition Changes

#### 2.5.1. Fe, Cu, Zn, and Mn

**Figure 4:**
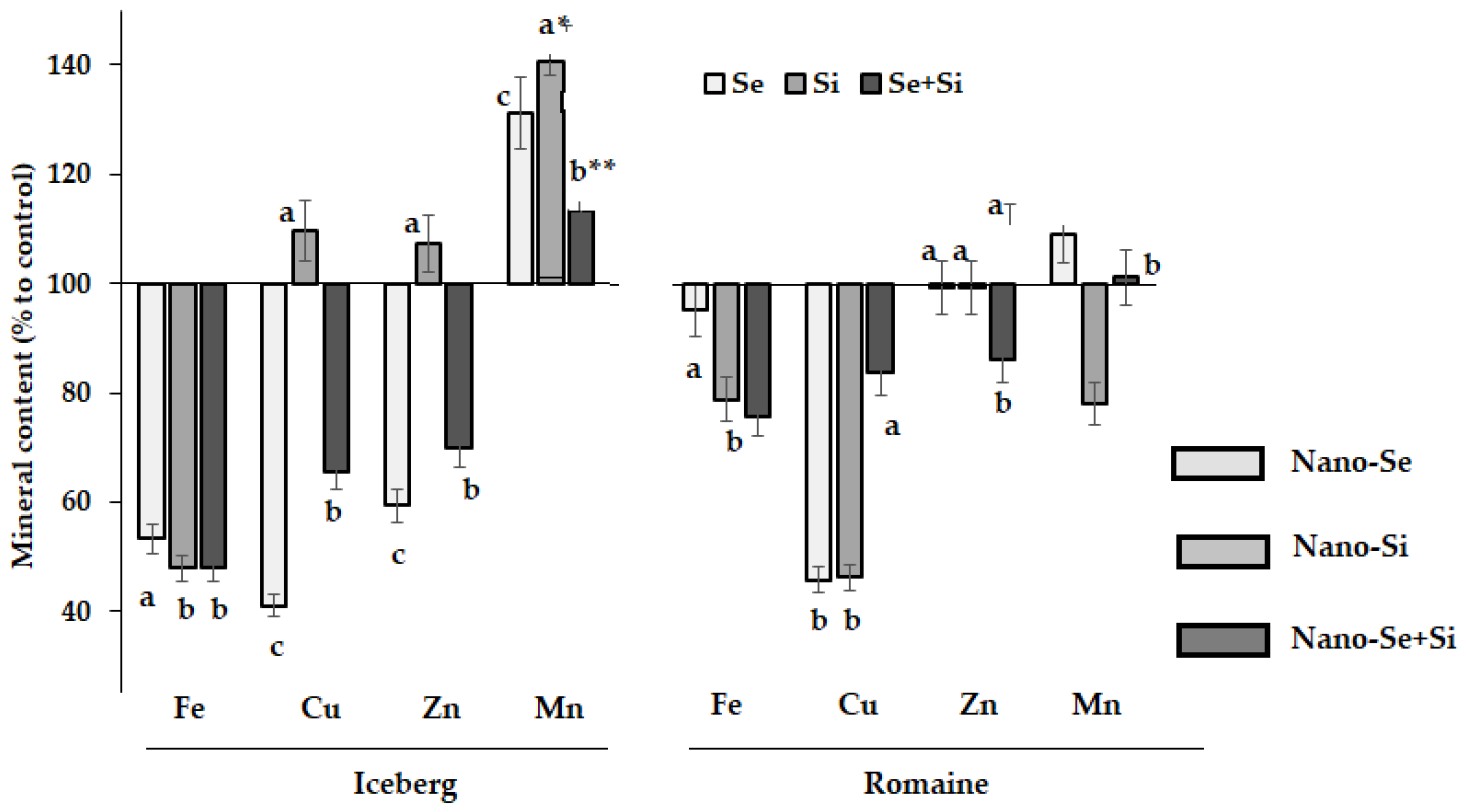
Changes in mineral content in Iceberg and Romaine lettuce leaves under nano-Se and/or nano-Si supply. (*): the value is decreased by 5-fold; (**): the value is decreased by 2-fold. For each element, values with the same letters do not differ statistically according to Duncan’s test at *p* < 0.05.

The obtained results of the lettuce mineral profile showed 3.0–5.3 times higher levels of Fe and a 1.9–5.8 times higher Cu content in Romaine leaves, compared to the Iceberg (Table 4 and Figure 4), along with 38.3 times higher concentrations of Mn. Only Zn did not show different levels in the two lettuce types, compared to control leaves. All these elements are very frequently connected with plant adaptations to environmental impact [44], due to their active participation in the antioxidant defense system, growth processes, respiration (Fe and Cu), photosynthesis (Fe, Cu, and Mn), and transcription (Zn) [45].

Se/Si application decreased Fe content significantly in both cultivars, in accordance with the observations of Becker et al. [46], who reported that Si addition reduced Fe concentration in rice in the absence of significant stress, upregulating the Fe homeostasis-related genes in roots. A significant drop in Fe accumulation was also recorded in lettuce plants subjected to Se treatment [47].

Based on the literature reports, the main effect of Se/Si treatment on plant mineral composition occurs via Se/Si interaction with soil components, affecting nutrient availability, pH, and rhizosphere microorganisms [48]. The two latter factors become inapplicable in a closed hydroponic system and under foliar Se/Si supply; therefore, it is only possible to monitor nutrient bioavailability, genetic variation, and the relationship between the two microelements and biologically active compounds within a species. The latter compounds are represented by phytohormones, specific antioxidants (ascorbic acid and polyphenols), and changes in the activity of plant enzymes, especially those containing the above-mentioned elements in their active centers. Previous controversial results regarding Se- [48] and Si-treated [49] plants, and scant information about the Se/Si nanoparticle effect, promote a special interest in the interaction between the nano-Se/Si and the microelements Fe, Mn, Cu, and Zn in the two lettuce types, Iceberg and Romaine, which greatly differ in their initial content of these microelements.

Indeed, in Iceberg lettuce, Fe content ranged from 47.9 to 53.4% of the control value, compared to 75.8–95.1% in Romaine. The corresponding variation in Cu was 41.0–109.6% in Iceberg, compared to 46.2–83.7% in Romaine; for Zn it was 50.4–107.3%, compared to 86.1–99.2%; and the highest differences were recorded for Mn: 131.2–705% and 89.0–109.1%, respectively. All treatments dramatically increased the Mn content in Iceberg, while a slight rise of 9.1% was recorded in Romaine plants, but only under nano-Se supply. Furthermore, nano-Si slightly increased the concentrations of Cu and Zn in Iceberg but not in Romaine leaves, even eliciting a significant Cu decrease.

As expected, in Iceberg (but not in Romaine), Mn increase has the highest importance among the factors affecting plant response to nano-Si/Se supply. In this respect, Mn guarantees the activity of various enzymes due to its presence in their active center (superoxide dismutase, catalase, and RNA polymerases, vital for plant growth) [50]; accordingly, it may be hypothesized that the detected beneficial effect of nano Se/Si supply to Iceberg lettuce is supposedly achieved both by enhancement of the Mn assimilation level and changes in the activity of Mn-containing enzymes, although specific investigations are necessary to confirm the hypothesis.

**Figure 5:**
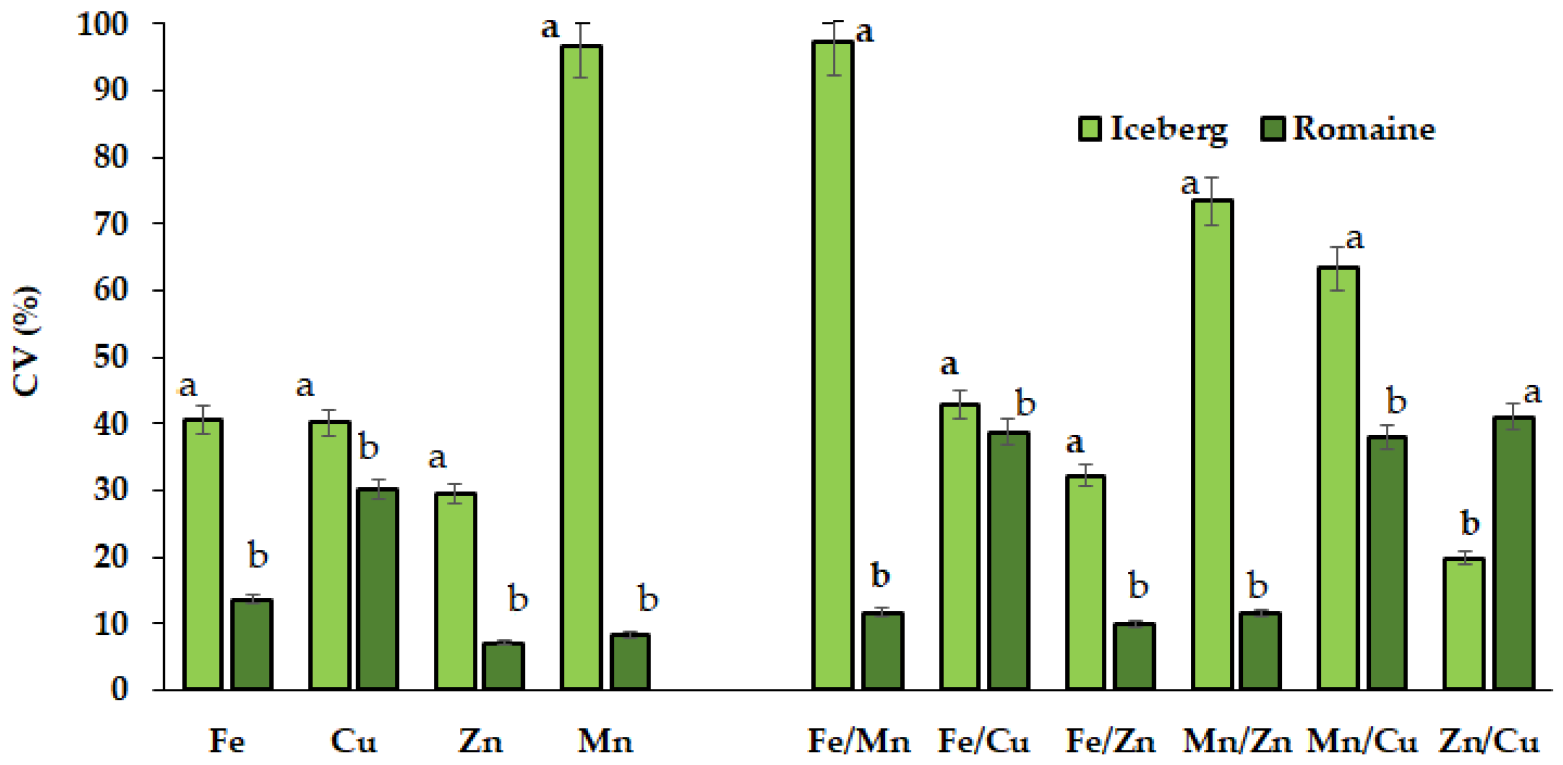
Coefficient of variation for Fe, Cu, Zn, and Mn, along with the ratios between the mentioned microelements in Iceberg and Romaine lettuce under nano-Se/Si treatment. For each parameter, values with the same letters do not differ statistically according to Duncan’s test at *p* < 0.05.

These peculiarities are clearly reflected in higher CV values calculated for Fe, Mn, Zn, Cu, and the mineral ratios in Iceberg lettuce, compared to Romaine, indicating major differences in adaptation mechanisms between the chosen lettuce types (Figure 5).

**Figure 6:**
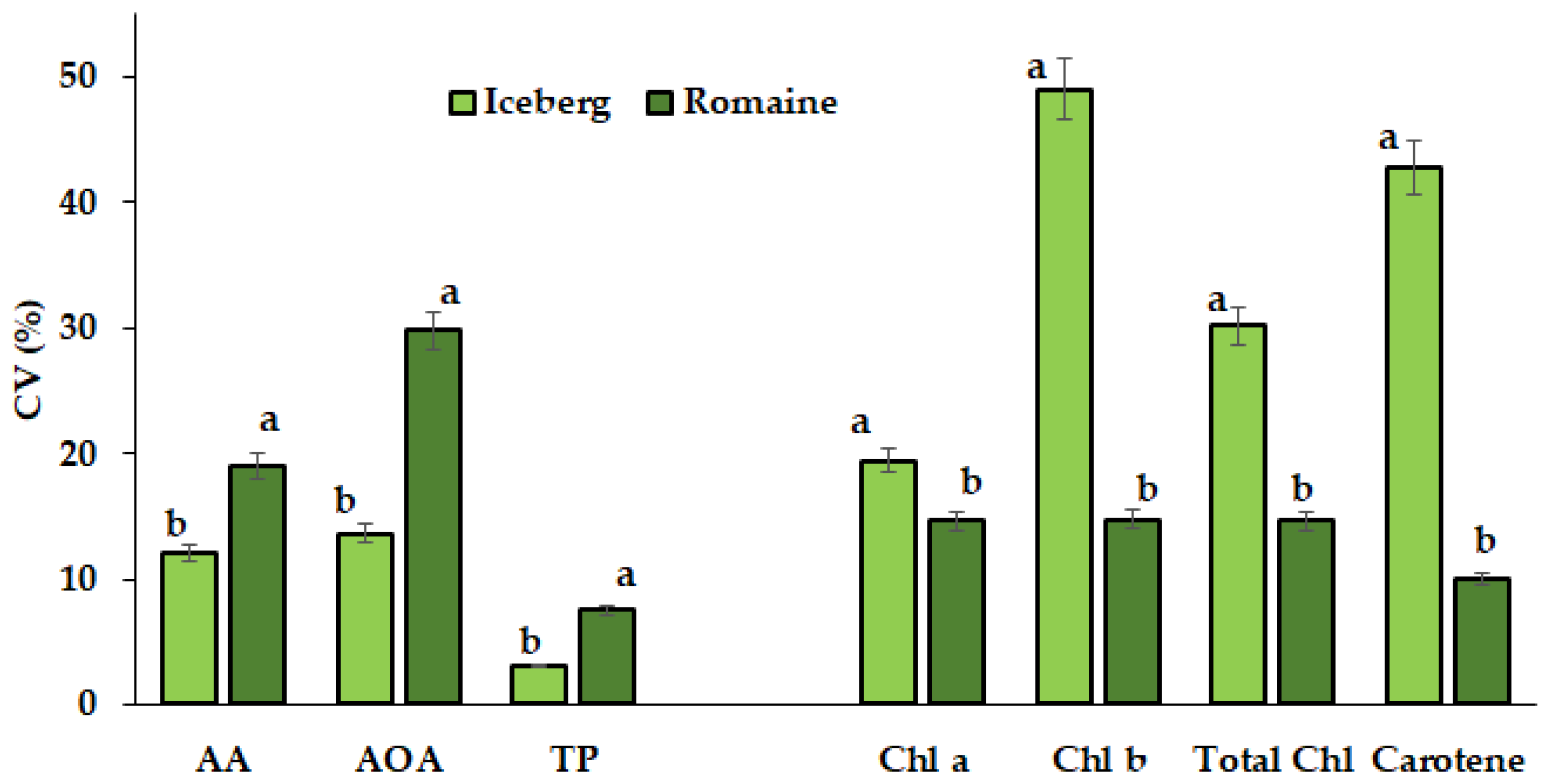
Coefficient of variation in the antioxidant parameters (AA, AOA, TP) and photosynthetic pigment accumulation (Chl and carotene). For each parameter, values with the same letters do not differ statistically according to Duncan’s test at *p* < 0.05. AA: ascorbic acid; AOA: total antioxidant activity; TP: total polyphenol content; Chl: chlorophyll.

Furthermore, significant variations in the mineral profile of Iceberg lettuce were accompanied by the appropriate CV differences in photosynthetic pigment levels and antioxidant parameters (Figure 6). Notably, the CV values of the antioxidant parameters (ascorbic acid, AOA, and TP) were significantly higher in the Romaine plants compared to Iceberg. These results indicate that Se/Si supply to Iceberg lettuce firstly affected photosynthesis and mineral profile, while in Romaine plants a more powerful effect of the mentioned treatments arose on plant antioxidant status.

Interestingly, the lower sensitivity of the Romaine lettuce to Se/Si treatment may relate to the initially higher levels of these microelements, diminishing the need for mineral level regulation. Nevertheless, both lettuce types showed similar Cu decrease under nano-Se supply, confirming their known antagonistic relationship [48,51].

The most dramatic Fe decrease was recorded in Iceberg lettuce under all treatments, contrary to the Romaine plants showing a significant reduction in this element only under single or joint Si supply (Table 4). Supposedly, Se and Si may affect Fe assimilation in Iceberg and, to a lesser extent, Romaine roots via regulation of the expression of genes participating in element transportation and accumulation [52], as well as an increase in cell wall thickness, thus reducing the element transfer across root cell membranes [53,54,55].

According to Cheng et al. [56], just the opposite effect of soil nano-Se application was recorded on the mineral profile of lettuce, increasing the content of most macro- and microelements. The explanation of the mentioned differences may relate to the beneficial effect of nano-Se on soil rhizo-microorganisms, thus improving nutrient bioavailability. The latter phenomenon was not recorded under Se foliar supply in hydroponic system and/or dependent on genetic peculiarities of the chosen lettuce type. Further investigations are necessary to examine the reasons for the detected variations.

#### 2.5.2. Selenium

Though the differences were not statistically significant in Se accumulation in Iceberg and Romaine plants, there was a tendency toward higher Se concentration in Romaine, either under single or joint Se/Si supply (Table 4). Under single Se and Si applications, both lettuce types provided 920–1030 µg Se kg^−1^ d.w., compared to lower values (611–720 µg kg^−1^ d.w.) associated with the joint treatment. These contents represent about 8–10% of RDA Se under consumption of 100 g of fresh lettuce. The lower Se accumulation ability of plants under joint nano-Se/Si supply was in accordance with the antagonistic relationship between these microelements recorded in the chosen conditions; they are consistent with the lack of significant changes in the total chlorophyll content and leaf dry biomass under joint Se/Si treatment.

### 2.6. Limitations and Practical Applicability

The present work refers to the beneficial effect of the foliar nano-particle (NP) application in hydroponics, applying only single concentrations of nano-Se (14 mg L^−1^) and nano-Si (7 mg L^−1^). Taking into account the previously detected effect of Se/Si ratio on the efficiency of NP supply [38] and the lack of corresponding data regarding the evaluation of application efficiency of ionic forms of the mentioned microelements, further investigations are needed to deepen the peculiarities of the NP technology utilization. From the practical point of view, the obtained data provide important information on the varietal differences between two distinct lettuce types in response to NP treatment, suggesting the possibility of a Se/Si antagonistic relationship and the two following major mechanisms of plant response (1) via predominant activation of the antioxidant status (modulating genes involved in secondary metabolite biosynthesis), and (2) via dramatic changes in the plant mineral profile affecting transport protein genes.

## 3. Material and Methods

### 3.1. Experimental Protocol and Design

*Lactuca sativa* L. plants were grown in the experimental hydroponic system at the Baikov Institute of Metallurgy and Material Science RAS (Moscow, Russia). Lettuce seeds (Romaine type, cv. Auvona RZ, and Iceberg type, cv. Lalique RZ), Rijk Zwaan production (Holland), were sown in rockwool cubes (2.5 cm × 2.5 cm × 3.0 cm) and placed in a plastic tray, watered, and kept in the dark at 25 °C for 3 days. After emergence, seedlings were subjected to artificial light and fertilized with a nutrient solution with an electric conductivity of 1.5 ± 0.1 mS cm^−1^ and a pH of 6.2 ± 0.1, whose composition is presented in Table 5.

**Figure 7:**
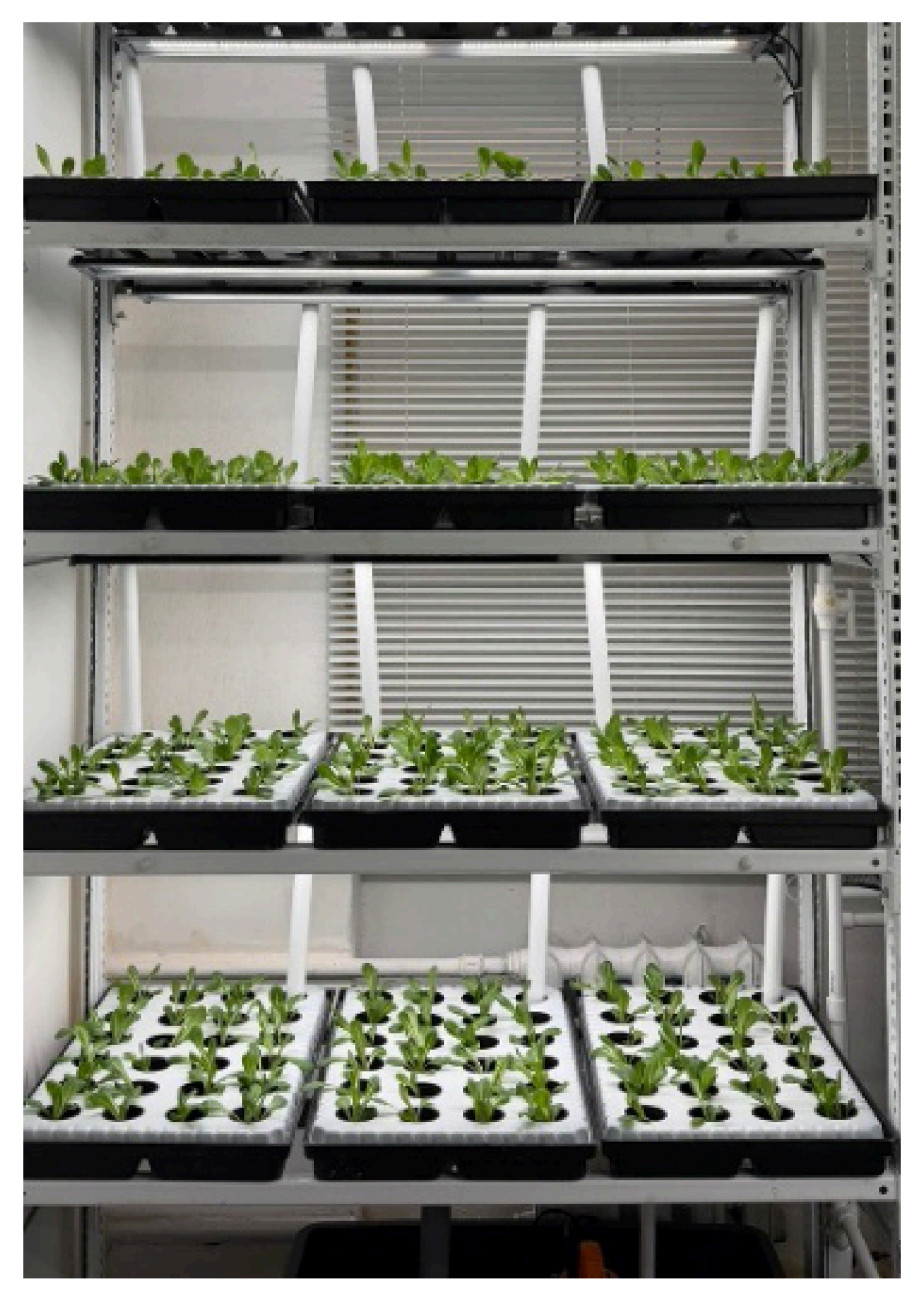
Overview of the hydroponic growing unit.

The hydroponic growing chamber was equipped with a DFT system (Deep Flow Technique) (Figure 7). Each tray contained 22 plants, spaced 10 cm apart within and between rows, with a light intensity (Photosynthetic Photon Flux Density, PPFD) of 346.8 ± 23.1 µM (m^−2^·c) and with 14 h of light daily (Led Farm 80.0.X; Led Optotechnologies, Minsk, Belarus). The nutrient solution (Table 5) was supplied for 15 min at 1 h intervals. Temperature and relative humidity were maintained at 18–22 °C and 53–57%, respectively.

The experimental protocol was based on the factorial combination of two lettuce types (Romaine and Iceberg) and three biofortification treatments (Nano-Se, Nano-Si, and Nano-Se+Si) plus an untreated control, using a split-plot design.

Nanoparticles were foliar-supplied twice according to the scheme presented in Table 6, and the control plants were sprayed with distilled water. The experiment was repeated twice in September and December.

The mentioned concentrations of nano-Se and nano-Si were chosen based on the results of previous investigations related to the high growth-promoting effect of nano-Si [22] and nano-Se [57].

After harvesting, determinations were made of growth indices. Half of the samples were stored, whereas the ascorbic acid content was measured on the remaining aliquot. The residue was dried at 70 °C for 24 h, homogenized, and used to determine the total antioxidant activity (AOA), total polyphenol content (TP), and mineral profile.

### 3.2. Chlorophyll and Carotene

The photosynthetic pigments were analyzed spectrophotometrically (spectrophotometer Unico 2804 UV, USA) on 96% ethanol extracts of fresh leaves [58]. Chlorophyll and carotene concentrations were calculated using the following equations:

Chl-a = 13.36A_664_ − 5.19A_649_;

Chl-b = 27.43A_649_ − 8.12A_664_;

C c = (1000A_470_ − 2.13 Chl-a − 87.63 Chl-b)/209;

where A = Absorbance; Chl-a = Chlorophyll a; Chl-b = Chlorophyll b; and C c = Carotene.

### 3.3. Ascorbic Acid

The ascorbic acid was determined in triplicate by visual titration of 6% trichloroacetic acid plant extracts with Tillman’s reagent [59].

### 3.4. Total Polyphenol Content (TP)

The Folin–Ciocalteu colorimetric method was used for the determination of the total polyphenol content (TP) on 70% ethanol extracts, as previously described [60]. To use the representative probe and diminish the possible negative effect of enzymes, the determination of polyphenols was carried out on homogenized dry leaves. The polyphenol extraction was made by heating dry leaf homogenates (0.5 g) in 70% ethanol at 80 °C for 1 h. After adjusting the sample volume to 25 mL and subsequent filtration, a reaction between polyphenols contained in the filtrate and Folin–Ciocalteu reagent was triggered in the presence of Na_2_CO_3_. One hour later, the absorbance of the reaction mixture at 730 nm was recorded by a spectrophotometer (Unico 2804 UV, Dayton, NJ, USA), and the concentration of polyphenols was calculated using 0.02% gallic acid as the external standard. The results are expressed as mg of Gallic Acid Equivalents per g of dry weight (mg GAE g^−1^ d.w.).

### 3.5. Antioxidant Activity (AOA)

The antioxidant activity was assessed using a redox titration method [42,59] based on a reaction of KMnO_4_ with the antioxidants present in 70% ethanolic extracts of dry samples, as described in Section 3.4. Gallic acid was used as an external standard, and the results are expressed in mg of Gallic Acid Equivalents (mg GAE g^−1^ d.w.).

### 3.6. Preparation and Characterization of Selenium Colloidal Solution

Nanoparticles of Se and Si were acquired using pulse laser ablation in deionized water, as previously reported [28,61]. The corresponding targets were subjected to nanosecond Nd:YAG laser irradiation with a wavelength of 1064 nm, pulse duration of 12 ns, and energy of 2.5 J per pulse, which was concentrated on the target using a lens.

The Se/Si nanoparticle concentration was analyzed via inductively coupled plasma atomic emission spectrometry (ICP-AES) using an ULTIMA 2 (Horiba Jobin Yvon, Palaiseau, France) spectrometer.

### 3.7. Selenium Content

Selenium was analyzed by the micro-fluorometric method (Fluorate 02-5M, Lumex, Saint Petersburg, Russia) [62], using the fluorescence value of a complex between Se^4+^ and 2,3-diaminonaphtalene (piazoselenol) in hexane after wet digestion of the samples with a mixture of nitric–perchloric acids and subsequent reduction of selenate (Se^6+^) to selenite (Se^4+^). Each determination was performed in triplicate. The precision of the results was verified using a reference standard, a lyophilized Mitsuba stem in each determination with a Se concentration of 1865 µg kg^−1^ (Federal Scientific Vegetable Center, Moscow region, Russia). The results are expressed in µg kg^−1^ d.w.

### 3.8. Fe, Mn, Cu, and Zn Content

Fe, Cu, Mn, and Zn concentrations in dried homogenized samples of lettuce leaves were determined in triplicate via Atomic Absorption Spectrometry (AAS) on a Shimadzu GFA-7000 spectrophotometer (Shimadzu, Kyoto, Japan) after wet sample digestion at 20–425 °C using concentrated HNO_3_ and H_2_SO_4,_ followed by subsequent dissolution of the residue in 3% HNO_3_ [63].

### 3.9. Lettuce Storage

After harvesting, portions of the lettuce leaves were immediately inserted into polyethylene bags, which were tightly closed and stored in a Camera Incubator DFI-80 (MRC-Laboratotory Instruments, Horlow, UK) under isothermal conditions (4 °C) and 75% relative humidity. The ascorbic acid content was measured on the 7th and 12th day of storage.

### 3.10. Statistical Analysis

The data were processed by one-way analysis of variance, and mean separations were performed through Duncan’s multiple range test, with reference to the 0.05 probability level, using SPSS software version 30 (IBM, Armonk, NY, USA). Data expressed as a percentage were subjected to angular transformation before processing.

## 4. Conclusions

The obtained results indicate that the nutritional density of the lettuce genotype greatly affects plant response to nano-Se/Si treatment, eliciting significant changes in photosynthetic pigment accumulation, chlorophyll a/b ratio, mineral profile, antioxidant parameters, and shelf-life. Single application of nano-Se or nano-Si had overall higher beneficial effects on both Iceberg and Romaine lettuce types, compared to joint foliar Se/Si supply. Higher variations in response to Se/Si treatments were recorded in Iceberg lettuce in terms of mineral profile and photosynthetic pigments, and in the Romaine type regarding the antioxidant parameters. Under all experimental treatments, Iceberg displayed the most significant Fe decrease and Mn increase, with the opposite effect in Romaine plants, which suggests important genetic peculiarities of plant responses to foliar supply of nano Se/Si. Further research is expected to reveal consistent patterns of Se/Si interaction with essential elements based on Se/Si chemical form, method of supply (foliar or nutrient solution), and concentration.

## Figures and Tables

**Table 1:** Yield and growth indices of lettuce.

Parameter	Control	Nano-Se	Nano-Si	Nano-(Se + Si)
Iceberg				
Plant fresh weight (g)	33.6 ± 1.1 b	40.3 ± 2.3 a	35.8 ± 1.2 b	33.6 ± 1.1 b
Plant dry weight (g)	1.51 ± 0.05 b	1.73 ± 0.09 a	1.60 ± 0.05 b	1.55 ± 0.05 b
Number of leaves	11.0 ± 1.0 b	14.0 ± 1.1 a	12.7 ± 1.7 c	14.8 ± 1.1 a
Leaf fresh weight (g)	3.05 ± 0.31 a	2.88 ± 0.25 a	3.06 ± 0.15 b	2.27 ± 0.22 b
Leaf dry matter (%)	4.5 ± 0.4 a	4.3 ± 0.3 a	4.2 ± 0.3 a	4.6 ± 0.4 a
Romaine				
Plant fresh weight (g)	36.1 ± 3.2 b	47.6 ± 4.6 a	44.9 ± 4.0 a	40.1 ± 4.0 ab
Plant dry weight (g)	2.02 ± 0.15 c	2.95 ± 0.27 a	2.69 ± 0.24 ab	2.33 ± 0.22 bc
Number of leaves	11.7 ± 1.0 b	12.1 ± 1.0 ab	12.8 ± 1.2 ab	14.2 ± 1.3 a
Leaf fresh weight (g)	3.10 ± 0.30 b	3.95 ± 0.40 a	3.21 ± 0.30 b	2.83 ± 0.20 b
Leaf dry matter (%)	5.6 ± 0.5 a	6.2 ± 0.6 a	6.0 ± 0.6 a	5.8 ± 0.5 a

Along each line, values with the same letters do not differ significantly according to Duncan’s test at *p* < 0.05.

**Table 2:** Photosynthetic pigments in Iceberg and Romaine lettuce leaves.

Parameters	Control	Nano-Se	Nano-Si	Nano-(Se+Si)
Iceberg				
Chl a (mg g^−1^ f.w.)	0.68 ± 0.04 b	0.77 ± 0.10 b	1.07 ± 0.04 a	0.70 ± 0.03 b
Chl b (mg g^−1^ f.w.)	0.31 ± 0.02 c	0.63 ± 0.06 b	0.86 ± 0.04 a	0.24 ± 0.02 d
Total chl (mg g^−1^ f.w.)	0.99 ± 0.08 c	1.40 ± 0.12 b	1.93 ± 0.16 a	0.94 ± 0.08 c
Carotene (mg g^−1^ f.w.)	0.08 ± 0.01 c	0.22 ± 0.02 a	0.20 ± 0.01 bc	0.11 ± 0.01 b
Chl a/chl b	2.19	1.22	1.24	2.92
Chl/carotene	12.4	6.4	19.3	8.5
Romaine				
Chl a (mg g^−1^ f.w.)	1.65 ± 0.12 bc	1.88 ± 0.15 ab	2.09 ± 0.20 a	1.40 ± 0.12 c
Chl b (mg g^−1^ f.w.)	1.01 ± 0.10 b	1.17 ± 0.10 a	1.34 ± 0.11 a	0.91 ± 0.08 b
Total chl (mg g^−1^ f.w.)	2.66 ± 0.22 bc	3.05 ± 0.29 ab	3.43 ± 0.31 a	2.31 ± 0.20 c
Carotene (mg g^−1^ f.w.)	0.29 ± 0.02 a	0.31 ± 0.03 a	0.31 ± 0.03 a	0.24 ± 0.02 b
Chl a/chl b	1.63	1.61	1.56	1.54
Chl/carotene	9.2	9.8	11.1	9.6

Chl: chlorophyll. Along each line, values with the same letters do not differ significantly according to Duncan’s test at *p* < 0.05.

**Table 3:** Antioxidant activity and polyphenol content in Iceberg and Romaine lettuce types.

Parameter	Control	Nano-Se	Nano-Si	Nano-(Se + Si)
Iceberg				
AA (mg 100 g^−1^ f.w.)	5.0 ± 0.3 c	6.0 ± 0.3 b	7.0 ± 0.4 a	6.5 ± 0.4 a
AOA (mg GAE g^−1^ d.w.)	24.5 ± 2.0 c	32.9 ± 2.9 ab	34.8 ± 3.0 a	27.7 ± 2.2 bc
TP (mg GAE g^−1^ d.w.)	15.7 ± 0.8 a	16.2 ± 1.1 a	16.9 ± 1.1 a	15.7 ± 1.0 a
Romaine				
AA (mg 100 g^−1^ f.w.)	9.0 ± 0.9 c	14.5 ± 1.1 a	12.0 ± 0.9 b	9.9 ± 0.8 c
AOA (mg GAE g^−1^ d.w.)	25.1 ± 2.1 b	55.0 ± 5.1 a	58.2 ± 5.2 a	64.1 ± 5.8 a
TP (mg GAE g^−1^ d.w.)	15.1 ± 1.5 a	17.8 ± 1.4 a	16.7 ± 1.3 ba	14.8 ± 1.3 a

AA: ascorbic acid; AOA: total antioxidant activity; TP: total polyphenol. Along each line, values with the same letters do not differ significantly according to Duncan’s test at *p* < 0.05.

**Table 4:** Mineral composition of Iceberg and Romaine lettuce leaves (mg kg^−1^ d.w.).

Element	Control	Nano-Se	Nano-Si	Nano-(Si + Se)
Iceberg				
Fe	37.8 ± 3.1 a	20.2 ± 1.9 b	18.1 ± 1.7 b	18.1 ± 1.7 b
Cu	2.49 ± 0.21 a	1.02 ± 0.10 c	2.73 ± 0.24 a	1.63 ± 0.14 b
Zn	12.30 ± 1.20 a	7.31 ± 0.71 b	13.20 ± 1.22 a	8.58 ± 0.84 b
Mn	1.38 ± 0.13 d	1.81 ± 0.17 c	9.73 ±0.94 a	3.14 ± 0.30 b
Se	0.045 ± 0.004 c	0.920 ± 0.088 a	0.047 ± 0.004 c	0.611 ± 0.059 b
Romaine				
Fe	112.5 ± 10.8 a	107.0 ± 10.0 a	88.6 ± 8.5 b	85.3 ± 8.3 b
Cu	11.35 ± 1.10 a	5.31 ± 0.52 c	5.25 ± 0.51 c	9.50 ± 0.93 b
Zn	12.20 ± 1.10 a	12.10 ± 1.20 a	12.10 ± 1.20 a	10.50 ± 1.00 a
Mn	53.01 ± 5.22 ab	57.82 ± 5.67 a	47.20 ± 4.34 a	53.60 ± 5.30 a
Se	0.035 ± 0.002 c	1.030 ± 0.008 a	0.026 ± 0.001 c	0.720 ± 0.006 b

Along each line, values with the same letters do not differ significantly according to Duncan’s test at *p* < 0.05.

**Table 5:** Composition of nutrient solution (mg L^−1^).

Macroelement	Concentration (mg L^−1^)	Microelement	Concentration (mg L^−1^)
Ammonium-nitrogen	25.9	Sulfur	37.8
Nitrate-nitrogen	189.7	Boron	0.49
Phosphorus	36.2	Copper	0.14
Potassium	335.4	Zinc	0.95
Magnesium	28.2	Iron	3.4
Calcium	95.0	Manganese	1.62
		Molybdenum	0.14

**Table 6:** Experiment description.

Experiment	Sowing Date	First Treatment Date and Nanoparticle Concentration	Second Treatment Date and Nanoparticle Concentration	Harvest Date
First	22 August 2025	10 September 2025 (nano-Se 25 mg L^−1^, nano-Si 14 mg L^−1^)	17 September 2025 (nano-Se 12.5 mg L^−1^, nano-Si 7 mg L^−1^)	29 September 2025
Second	14 November 2025	3 December 2025 (nano-Se 25 mg L^−1^, nano-Si 14 mg L^−1^)	10 December 2025 (nano-Se 12.5 mg L^−1^, nano-Si 7 mg L^−1^)	22 December 2025

## Data Availability

The datasets generated and/or analyzed during the current study are available from the corresponding author upon reasonable request.
